# Chinese expert consensus on Bruton tyrosine kinase inhibitors in the treatment of B-cell malignancies

**DOI:** 10.1186/s40164-023-00448-5

**Published:** 2023-10-16

**Authors:** Yuqin Song, Shang-Ju Wu, Zhixiang Shen, Donglu Zhao, Thomas Sau Yan Chan, Huiqiang Huang, Lugui Qiu, Jianyong Li, Tran-der Tan, Jun Zhu, Yongping Song, Wei-Han Huang, Weili Zhao, Herman Sung Yu Liu, Wei Xu, Naizhi Chen, Jun Ma, Cheng-Shyong Chang, Eric Wai Choi Tse

**Affiliations:** 1 Peking University Cancer Hospital and Institute, Beijing, China; 2 Hematology Division, Department of Internal Medicine, National Taiwan University Hospital, Taiwan, China; 3 Shanghai Jiaotong University Affiliated Ruijin Hospital, Shanghai, China; 4 Harbin Hematology and Oncology Institute, Heilongjiang, 150007 China; 5 Department of Medicine, Queen Mary Hospital, Hong Kong, China; 6 Sun Yat-Sen University Cancer Center, Guangdong, China; 7 Institute of Hematology and Blood Diseases Hospital, Tianjing, China; 8 Jiangsu Provincial People’s Hospital, Jiangsu, China; 9 Department of Hematology and Medical Oncology, Koo Foundation Sun Yat-Sen Cancer Center, Taiwan, China; 10 The First Affiliated Hospital of Zhengzhou University, Henan, China; 11 Department of Clinical Pathology, Hualien Tzu Chi Hospital, Buddhist Tzu Chi Medical Foundation, Taiwan, China; 12 Premier Medical Centre, Hong Kong, China; 13 Macau Society of Hematology and Oncology, Macau, China; 14 Division of Hematology-Oncology, Department of Internal Medicine, Chang Bing Show Chwan Memorial Hospital, Taiwan, China; 15 Department of Healthcare Administration, Central Taiwan University of Science and Technology, Taiwan, China; 16 Department of Medicine, School of Clinical Medicine, University of Hong Kong, Hong Kong, China

**Keywords:** BTK inhibitors, China, Consensus, Non-Hodgkin lymphoma, Targeted therapy

## Abstract

Targeted therapy with Bruton tyrosine kinase (BTK) inhibitors have revolutionized the treatment of patients with various B-cell malignancies. BTK inhibitors such as ibrutinib, zanubrutinib, orelabrutinib, and acalabrutinib have shown good clinical efficacy and better safety profiles than those of traditional chemotherapy and chemoimmunotherapy regimens. Multiple studies on new BTK inhibitors are ongoing, which may provide more therapeutic options for the treatment of B-cell malignancies. Considering the unmet need of evidence on BTK inhibitors in all clinical settings and to standardize the use of BTK inhibitors available in mainland China, Taiwan, Hong Kong, and Macau regions, this consensus has been formulated for the treatment of various B-cell malignancies based on the clinical practice and available evidences on the use of BTK inhibitors. The recommendations of this consensus will provide guidance to physicians and clinical researchers on the effective treatment of B-cell malignancies with BTK inhibitors.

**Supplementary Information:**

The online version contains supplementary material available at 10.1186/s40164-023-00448-5.

## Background

B-cell malignancies comprise the most common hematologic malignancy, which is categorized as Hodgkin lymphoma (HL) and non-Hodgkin lymphoma (NHL) [1]. The GLOBOCAN 2022 estimated 76,510 and 8240 cases of NHL and HL, respectively, in the United States while a much larger proportion of NHL and HL cases (97,788 and 6984, respectively) were estimated in China [2]. The high prevalence of B-cell malignancies demands an effective treatment and management options. With the advancement in tumor biology and modernization of biomedical technology, molecular-targeted therapy has gained attention for the treatment of hematologic malignancies [3]. Bruton's tyrosine kinase (BTK) is a key effector molecule in B-cell development and is expressed in B-cell lymphomas [4]. Hence, targeting BTK to develop new treatment modalities for B-cell malignancies is appealing. Multiple studies have confirmed the therapeutic value of BTK inhibitors for a variety of B-cell malignancies [5]. Based on the clinical study data, the current National Comprehensive Cancer Network (NCCN^®^) guidelines recommend the use of BTK inhibitors in B-cell malignancies [6]. A Chinese version of the expert consensus on BTK inhibitors for the treatment of B-cell malignant tumors has been published in the *Journal of Leukemia & Lymphoma* in 2022 [7]. Ibrutinib was the first approved BTK inhibitor in the United States and China [8]. However, the off-target kinase inhibition by ibrutinib is associated with adverse events (AEs), which led to the development of more selective new-generation BTK inhibitors [9], such as, acalabrutinib, zanubrutinib, orelabrutinib, and tirabrutinib. Pirtobrutinib, a non-covalent BTK inhibitor, has also been approved by the Food and Drug Administration (FDA) [10], but it has not been approved in China yet. The approval information is listed in Table 1.

**Table 1:** BTK inhibitors for hematologic malignancies

BTK inhibitor	Indication: FDA approval	Indication: China approval
Ibrutinib	CLL/SLL, WM, R/R MZL, R/R MCL	China: CLL/SLL, R/R MCL, WM Hong Kong: CLL/SLL, R/R MCL, WM Taiwan: CLL/SLL, R/R MCL, WM, R/R MZL
Acalabrutinib	CLL/SLL, R/R MCL	China: R/R MCL; R/R CLL/SLL Hong Kong: CLL/SLL, R/R MCL Taiwan: CLL/SLL, R/R MCL
Zanubrutinib	CLL/SLL, WM, R/R MCL, R/R MZL	China: CLL/SLL, WM, R/R MCL Hong Kong: CLL/SLL, WM, R/R MCL Taiwan: CLL/SLL, WM, R/R MCL, R/R MZL
Orelabrutinib	None	China: R/R MCL, R/R CLL, R/R MZL
Tirabrutinib	R/R PCNSL	Taiwan: R/R PCNSL
Pirtobrutinib	R/R MCL	None

Cutoff date: September 2023

In 2023, ibrutinib voluntarily withdraws the indication for MCL in patients who have received at least 1 prior therapy and for MZL in patients who require systemic therapy and have received at least 1 prior anti-CD20-based therapy, due to requirements related to accelerated approval status granted by the FDA

BTK, Bruton tyrosine kinase; CLL, chronic lymphocytic leukemia; FDA, Food and Drug Administration; MCL, mantle cell lymphoma; MZL, marginal zone B-cell lymphoma; PCNSL, primary central nervous system lymphoma; R/R; relapsed/refractory; SLL, small lymphocytic lymphoma; WM, Waldenström macroglobulinemia

The evidence on the beneficial effects of BTK inhibitors could not address all clinical settings. In order to standardize the use of BTK inhibitors available in mainland China, Taiwan, Hong Kong, and Macau regions and benefit more patients with B-cell malignancies, the specialists of these regions collaborated and formulated the consensus based on the available evidence. This consensus considered the recent evidence as well and provided recommendations based on the level of evidence. The recommendations of this consensus will provide guidance to physicians and clinical researchers on the effective treatment of B-cell malignant tumors with BTK inhibitors.

### Methodology

In January 2023, a panel of discussion consisting of medical specialists and experts in clinical research was conducted. The methodology chosen to develop the consensus report was similar to the Nominal Group Technique but not exactly the same. The members in the review committee were from diverse geographic regions in Mainland China, Taiwan, Hong Kong, and Macau and diverse society of hematology. For constituting the committee experts, we approached the hematology societies located in these regions and got recommendations for experts from them. Also, a peer recommendation was taken into consideration while inviting committee panel review members. After finalizing the members of the review committee, we raised the questions that needed to be discussed in the kick-off meeting—to assign recommendations and evidence levels for the use of BTK inhibitors in different B-cell malignancies. The core activity of the review committee was to develop a consensus through systematic review, evidence synthesis, and inputs from practitioners. Evidence for this consensus was selected and reviewed by all committee members of the *Chinese Society of Clinical Oncology* (CSCO) and hematologists from Taiwan, Hong Kong, and Macau. The existing scientific evidence on BTK inhibitors in the treatment of B-cell malignant tumor was critically reviewed and discussed to formulate recommendations by the consensus members. Based on the quality of existing evidence and the strength of recommendations, the categories of consensus statements were divided in accordance with the NCCN definitions for scientific evidence and recommendations [6] (Table 2). The experts carefully assessed the quality of the cited studies and graded the consensus statements. After three rounds of consensus meetings, the drafted recommendations were rated by the consensus members through an email questionnaire (Additional file 1: Table S1) and then finalized in the summary meeting. The anonymous voting results are also listed followed by each recommendation.

**Table 2:** Strength of recommendations

Category	Description
1	Based upon high-level evidence, there is uniform consensus that the intervention is appropriate
2A	Based upon low-level evidence, there is uniform consensus that the intervention is appropriate
2B	Based upon low-level evidence, there is consensus that the intervention is appropriate
3	Based upon any level of evidence, there is a major disagreement that the intervention is appropriate

For the “uniform consensus” defined in category 1 and category 2A, a majority panel vote of at least 85% is required. For the consensus, defined in category 2B, a panel vote of at least 50% (but < 85%) is required. Lastly, for recommendations where there is a strong panel disagreement regardless of the quality of the evidence, it requires a panel vote of at least 25% to include and designate a recommendation as category 3

Evidence was selected for inclusion if they met the following criteria: clinical trials using BTK inhibitor monotherapy or in combination with chemoimmunotherapy and registered with the Clinialtrial.gov or Chinese Clinical Trial Registry (ChiCTR); inclusion of adult patients with B-cell malignancies of any type, at any stage, and any histology; and the evaluation of survival outcomes, disease control, response rate, quality of life, or toxicity. Retrospective studies were also considered if they fulfilled the abovementioned interventions and outcomes criteria. Studies conducted exclusively on patients with immunological disease or non-hematologic malignancies treated with BTK inhibitors were not considered.

### Mechanism of action

**Fig. 1:**
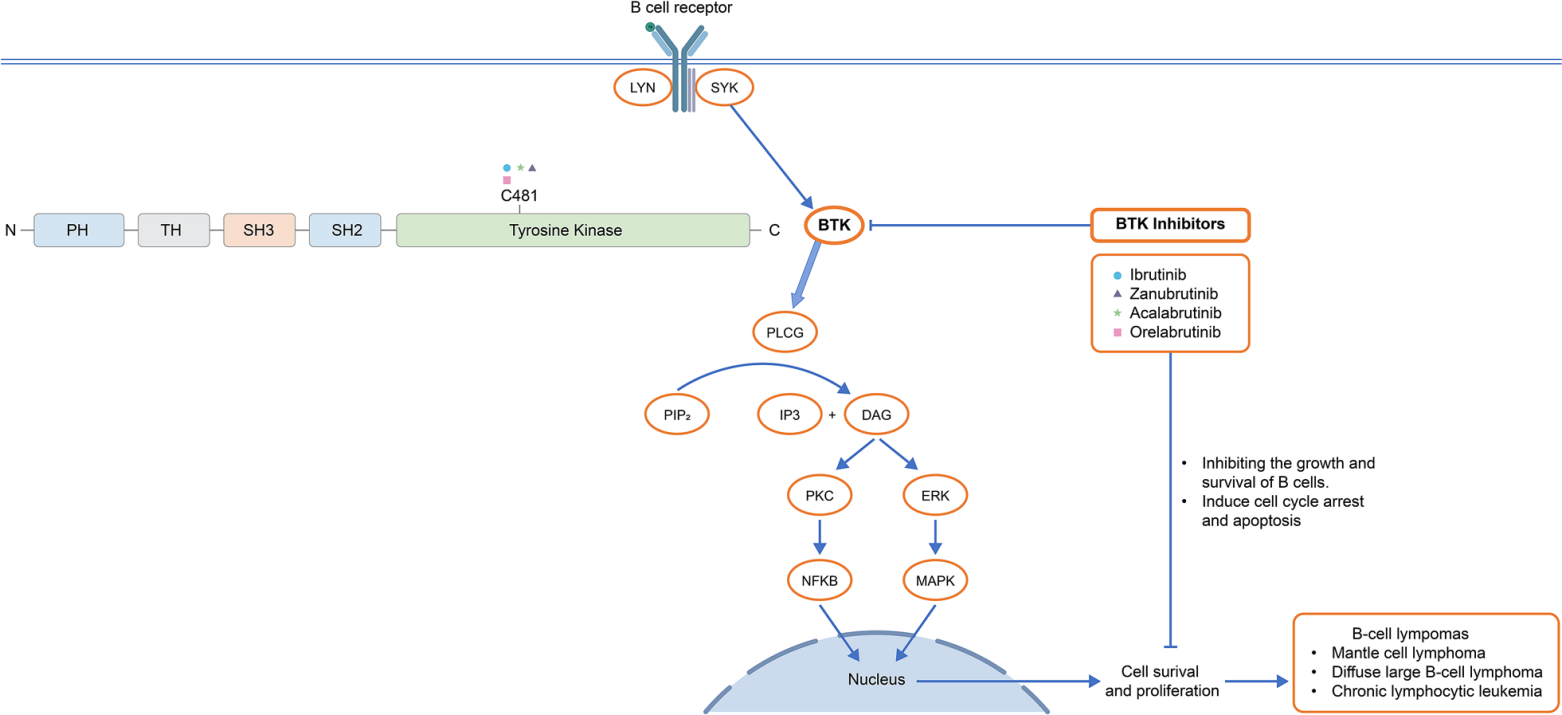
Mechanism of action of BTK inhibitors

BTK inhibitors target BTK, a protein involved in signaling pathways of various immune cells, including B cells and macrophages. BTK inhibitors covalently bind to a cysteine residue at position 481 in the adenosine triphosphate (ATP) binding site of BTK, irreversibly inhibiting its kinase activity [11, 12]. This binding prevents the transfer of phosphate groups from ATP to downstream signaling proteins, ultimately disrupting B-cell receptor signaling and inducing apoptosis in malignant B cells [13–17]. The mechanism of action of BTK inhibitors is presented in Fig. 1. BTK inhibitors represent an important and effective class of drugs for the treatment of B-cell malignancies [13].

### Pharmacokinetics and pharmacodynamics

BTK inhibitors are rapidly absorbed after oral administration with a median time to the peak concentration (T_max_) of 1–2 h [18]. Zanubrutinib and acalabrutinib can be administered without food, as food did not result in significant effects on the area under the curve (AUC) for these drugs [19, 20], whereas administration with food significantly increased the AUC of ibrutinib by twofold, compared with administration following an overnight fast [21]. BTK inhibitors undergo extensive metabolism, primarily via a CYP3A-mediated pathway [19–21]. It is suggested to avoid acalabrutinib and ibrutinib in patients with severe hepatic impairment, whereas zanubrutinib requires dosage modification in this patient population [22]. Moreover, co-administration of acalabrutinib with proton pump inhibitors should be avoided [23].

BTK inhibitors have the ability to cross the blood–brain barrier (BBB); therefore, these can be considered as a treatment option for patients with primary central nervous system lymphoma (CNSL) [24]. A proof-of-concept phase Ib study demonstrated a low cerebrospinal fluid (CSF) penetration of ibrutinib, as the median (range) AUC_0–24 nM.h_ in plasma was 977 (327–1562), whereas it was only 7.7 (2.21–16.5) in the CSF (CSF to plasma ratio: 0.78%). Furthermore, at a dose of 840 mg, the median (range) of 4 (0–24) h of the time above its enzymatic half-maximal inhibitory concentration (IC_50_; 0.5 nM) was observed in the CSF [25]. In a case series, the assessment of CSF distribution of zanubrutinib showed an excellent capability of zanubrutinib to cross the BBB. The mean plasma and CSF concentration of zanubrutinib was 143,190.6 ± 93,302.7 and 2941.1 ± 2382.01 pg/mL, respectively, with a median CSF/plasma ratio of 2.39% ± 1.71%, and the corrected CSF/plasma ratio after considering the high protein binding rate (94%) was 42.7% ± 27.7% (range, 8.6%–106.3%). Moreover, 95.7% of the samples had peak CSF concentrations of zanubrutinib above the enzymatic IC_50_ values [26].

Zanubrutinib and acalabrutinib are more selective than ibrutinib against off-target kinases [27, 28]. The results from kinase inhibition and cell-based assays demonstrated that zanubrutinib exhibited higher selectivity than ibrutinib, acalabrutinib, and acalabrutinib and its major metabolite (M27), with a selectivity comparable with orelabrutinib, by kinase profiling [29].

BTK resynthesis was faster in patients with chronic lymphocytic leukemia (CLL) than in healthy volunteers; therefore, it is hypothesized that complete/sustained BTK occupancy may improve the efficacy outcomes [27]. Zanubrutinib has exposure coverage above its IC_50_ during the entire dose interval for both twice-daily and once-daily dosing schedules with a high ratio of C_trough_/IC_50,_ resulting in significantly higher concentrations than IC_50_ during the entire 24 h dosing period for both dosing schedules [30]. However, the corresponding ratios of C_trough_/IC_50_ for ibrutinib and acalabrutinib were estimated to be consistently lower than 1, even after considering the active metabolites [11, 31].

## Clinical recommendations for BTK inhibitors in the treatment of malignant lymphoma

### Chronic lymphocytic leukemia (CLL)/small lymphocytic lymphoma (SLL)

CLL/SLL is characterized by the presence of monoclonal B-cell population in the peripheral blood [32]. The B-cell receptor activation plays an important role in the pathogenesis of CLL [33]. Therefore, the targeted inhibition of BTK can effectively treat CLL. With the advent of BTK inhibitors, the treatment armamentarium of CLL has expanded, offering an effective and well-tolerated therapeutic option in both treatment-naïve (TN) and relapsed/refractory (R/R) settings [34–37]. BTK inhibitors are recommended for the treatment of patients with CLL/SLL regardless of age, fitness, deletion of chromosome 17 (del [17p]) and/or TP53 mutation, and immunoglobulin heavy-chain variable-region (IGHV) gene mutational status. Multiple phase III studies have compared the efficacy and safety profile of new-generation BTK inhibitors (zanubrutinib and acalabrutinib) with the first-generation BTK inhibitor, ibrutinib. However, till date, head-to-head comparison of orelabrutinib with first-generation BTK inhibitor is not available and further research is warranted to demonstrate the benefit of orelabrutinib over the first-generation BTK inhibitor.

#### Treatment-naïve patients with CLL/SLL

Previously untreated patients with CLL/SLL have shown superior efficacy with BTK inhibitors than chemoimmunotherapy regimens [38]. TN patients with or without del(17p) and/or TP53 mutation have benefited from BTK inhibitors. Moreover, patients with and without IGHV mutation have also shown efficacy with BTK inhibitors over chemoimmunotherapy regimens. Currently, targeted therapy with BTK inhibitors has gained popularity as the most efficacious and safe treatment option for patients with CLL/SLL [39]. Few studies have demonstrated efficacy and tolerable safety profile of BTK inhibitors even in the elderly population [40]. The evidence of studies on BTK inhibitors in patients with CLL/SLL has been summarized in Table 3.

**Table 3:** Evidence of BTK inhibitors in patients with CLL/SLL

Patients type	Treatment regimen	Efficacy	Study	Phase	Patients (N)
Patients with treatment naïve CLL/SLL					
Without del(17p) and TP53 mutation	IR vs. FCR (fludarabine 25 mg/m^2^, cyclophosphamide 50 mg/m^2^, rituximab 50 mg/m^2^)	Statistically significant improvement in PFS and OS in the IR group compared with the FCR group (HR: 0.35; *P* < 0.001 and HR: 0.17; *P* < 0.001, respectively)	E1912 [41]	3	354 vs. 175
	Ibrutinib vs. chlorambucil	PFS: median-NR vs. 15 months; 59% vs. 9% (HR: 0.154; 95% CI: 0.108–0.220) 7-year OS: median NR vs. 89 months; 78% in ibrutinib (HR: 0.453; 95% CI: 0.276–0.743) ORR: 92% vs. 37%	RESONATE-2 [42]	3	136 vs. 133
	Zanubrutinib vs. BR	Better PFS improvement with zanubrutinib than BR regimen; 2-year PFS rate: 85.5% (HR: 0.42; 95% CI: 0·28–0·63; *P* < 0.0001)	SEQUOIA [38]	3	241 vs. 238
	BR vs. ibrutinib monotherapy vs. IR	2-year PFS rate: 74% vs. 87% (HR: 0.39; 95% CI: 0.26–0.58; *P* < 0.001) vs. 88% (HR: 0.38; 95% CI, 0.25–0.59; *P* < 0.001)	Alliance [43]	3	182 vs. 182 vs. 183
	Ibrutinib plus obinutuzumab vs chlorambucil (0.5 mg/kg bodyweight on days 1 and 15 of each 28-day cycle) plus obinutuzumab (100 mg on day 1, 900 mg on day 2, 1000 mg on day 8, and 1000 mg on day 15 of cycle 1, then 1000 mg on day 1 of each 28-day cycle for cycles 2–6)	30 months PFS: 79% vs. 31% Median PFS: HR: 0·23; 95% CI: 0·15–0·37; *P* < 0.0001	iLLUMINATE [44]	3	113 vs. 116
	Acalabrutinib + obinutuzumab vs. acalabrutinib monotherapy vs. obinutuzumab (day 1: 100 mg, 2: 900 mg, 8: 1000 mg, and 15: 1000 mg of cycle 1 and on day 1: 1000 mg of cycles 2–6) + chlorambucil (0.5 mg/kg on days 1 and 15 of each cycle)	Median PFS at 48-month follow-up: NR vs. NR vs. 17.5 months; PFS rate: 87% vs. 77.9% vs. 25.1%; *P* < 0.0001, *P* < 0.0001, and *P* = 0.0296 OS rate: 92.9% vs. 87.6% vs. 88.0%; *P* = 0.064, *P* = 0.9164, *P* = 0.0836	ELEVATE-TN [51]	3	179 vs. 179 vs. 177
With del(17p) and TP53 mutation	IR vs. Ibrutinib monotherapy vs. BR	Median PFS: NR vs. NR vs. 7 months	Alliance [43]	3	11 vs. 9 vs. 14
	Zanubrutinib	ORR: 94.5%; 18-month PFS rate: 90.6%; 18-month OS rate: 95.1%	SEQUOIA [38]	3	109
	Acalabrutinib	48-month PFS rate: 74.8%	ELEVATE-TN [51]	3	25
IGHV unmutated	IR vs. FCR	5-year PFS rates: 75% vs. 33%	E1912 [41]	3	210 vs. 71
	Acalabrutinib + obinutuzumab vs. acalabrutinib monotherapy vs. obinutuzumab + chlorambucil	48-month PFS rates: 77.1% vs. 85.7% (*P* < 0.0001 for A + O vs. O + C, *P* < 0.0001 for A vs. O + C)	ELEVATE-TN [51]	3	103 vs. 119 vs. 116
	Zanubrutinib vs. BR	Longer PFS with zanubrutinib than with BR (HR: 0.24; 95% CI: 0.13–0.43)	SEQUOIA [38]	3	125 vs. 121
IGHV mutation	IR vs. FCR	5-year PFS rates: 83% vs. 68%	E1912 [172]	3	70 vs. 44
	Acalabrutinib + obinutuzumab vs. acalabrutinib monotherapy vs. obinutuzumab + chlorambucil	48-month PFS rates: 87% vs. 77.9% vs. 25.1% (*P* = 0.0012 for A + O vs. O + C, *P* = 0.0551 for A vs. O + C)	ELEVATE-TN [51]	3	58 vs. 74
	Zanubrutinib vs. BR	Longer PFS with zanubrutinib than with BR (HR: 0.35; 95% CI: 0.19–0.64)	SEQUOIA [38]	3	109
Patients with R/R CLL/SLL					
Without del(17p) and TP53 mutation	Acalabrutinib vs. idelalisib + rituximab or BR	42-month PFS rate: 63% vs. 21%; HR: 0.30; 95% CI: 0.20–0.44; *P* < 0.0001	ASCEND [58]	3	155 vs. 155
	Zanubrutinib vs ibrutinib	24-month PFS rate: 78.4% vs. 65.9%; HR: 0.65; 95% CI: 0.49–0.86; *P* = 0.002	ALPINE [55]	3	207 vs. 208
With del(17p) and TP53 mutation	Acalabrutinib vs. ibrutinib	Median PFS of 38.4 months in both the groups	ELEVATE-RR [54]	3	268 vs. 265
	Zanubrutinib vs. ibrutinib	24-month PFS: 72.6% vs. 54.6%; HR: 0.53; 95% CI: 0.31–0.88	ALPINE [55]	3	75 vs. 75
	Acalabrutinib vs. idelalisib + rituximab or BR	42-month PFS: 57% vs. 10%; HR: 0.22; 95% CI: 0.12–0.39; *P* < 0.001	ASCEND [58]	3	22 vs. 13
	Ibrutinib vs. ofatumumab	HR: 0.175; 95% CI: 0.115–0.258	RESONATE [53]	3	147
IGHV mutated	Acalabrutinib vs. idelalisib + rituximab or BR	68% vs. 36%, HR: 0.34; 95% CI: 0.13–0.93; *P* = 0.027	ASCEND [58]	3	21
	Ibrutinib vs. ofatumumab	HR: 0.103; 95% CI: 0.067–0.159	RESONATE [53]	3	181
IGHV unmutated	Acalabrutinib vs. idelalisib + rituximab or BR	59% vs. 17%, HR: 0.29; 95% CI: 0.20–0.41; *P* < 0.001	ASCEND [58]	3	109 vs. 119
	Zanubrutinib vs. ibrutinib	HR: 0.64; 95% CI: 0.47–0.87	ALPINE [55]	3	72 vs. 98
	Ibrutinib vs. ofatumumab	HR: 0.200; 95% CI: 0.113–0.353	RESONATE [53]	3	85

A, acalabrutinib; BR, bendamustine plus rituximab; BTK, Bruton tyrosine kinase; C, chlorambucil; CI, confidence interval; CLL, chronic lymphocytic leukemia; FCR, fludarabine, cyclophosphamide, rituximab; IR, ibrutinib + rituximab; HR, hazard ratio; IGHV, immunoglobulin heavy-chain variable-region; MZL, marginal zone B-cell lymphoma; NR, not reached; PFS, progression-free survival; SLL, small lymphocytic lymphoma; TN, treatment naïve; O, obinutuzumab; ORR, overall response rate; OS, overall survival

#### Treatment-naïve patients with CLL/SLL with or without del(17p) and/or TP53 mutation

Patients without del(17p) have shown superior efficacy with BTK inhibitors than with chemoimmunotherapy in various studies. In the phase III ECOG-ACRIN E1912 study, younger patients without del(17p) received either ibrutinib plus rituximab (50 mg/m^2^ of body surface area on day 1 of cycle 2; 325 mg/m^2^ on day 2 of cycle 2; and 500 mg/m^2^ on day 1 of cycles 3 through 7) or chemoimmunotherapy with rituximab (50 mg/m^2^), fludarabine (25 mg/m^2^), and cyclophosphamide (50 mg/m^2^) (FCR). After a median follow-up of 34 months, the improvement in progression-free survival (PFS) and overall survival (OS) of the ibrutinib plus rituximab was statistically significant (*P* < 0.001) when compared with the FCR regimen [41]. In the RESONATE-2 study involving patients aged > 65 years, comparison of ibrutinib with chlorambucil showed significantly better PFS, OS, and overall response rate (ORR) in TN patients with CLL without del(17p) after a long-term follow-up [42]. Similarly, in the SEQUOIA study, zanubrutinib was associated with a significantly better PFS than bendamustine plus rituximab (BR) regimen (hazard ratio [HR]: 0.42, 95% confidence interval [CI]: 0.28–0.63, *P* < 0.0001) [38]. The results of the Alliance study showed that the ibrutinib monotherapy and ibrutinib plus rituximab had significantly better PFS than the BR regimen [43]. Moreover, the iLLUMINATE study observed that patients could benefit by the addition of BTK inhibitors in the monoclonal antibody regimen, as significantly longer PFS was obtained with ibrutinib plus obinutuzumab than chlorambucil (0.5 mg /kg on days 1 and 15 of each 28-day cycle) plus obinutuzumab (100 mg on day 1, 900 mg on day 2, 1000 mg on day 8, and 1000 mg on day 15 of cycle 1, then 1000 mg on day 1 of each 28-day cycle for cycles 2–6) [44]. Furthermore, in the ELEVATE‑TN study, elderly patients randomly received acalabrutinib and obinutuzumab, acalabrutinib monotherapy, or obinutuzumab (100 mg on day 1, 900 mg on day 2, 1000 mg on day 8, and 1000 mg on day 15 of cycle 2 and on day 1 [1000 mg] of cycles 2–6) and chlorambucil (0.5 mg/kg on days 1 and 15 of each cycle) and showed that acalabrutinib as monotherapy or in combination with obinutuzumab was associated with significantly longer PFS [45].

The patients with mutation of the TP53 tumor suppressor gene or the deletion of chromosome 17p (del[17p]) where TP53 is encoded, are high-risk patients with TP53 mutation and del(17p) influencing the prognosis of patients with CLL [46]. Studies have shown that patients with del(17p) or a mutation of TP53 have poor response to initial chemoimmunotherapy or are prone to relapse after remission, and BTK inhibitors are considered as the first choice of treatment for these patient populations [47]. For patients with del(17p) in the Alliance study, PFS was longer in patients receiving ibrutinib when compared with those in the BR group [43]. The SEQUOIA study included patients with del(17p) who were treated with zanubrutinib monotherapy, and the observed ORR, PFS, and OS rates were 94.5%, 90.6%, and 95.1%, respectively [38]. In the ELEVATE-TN study, patients with del(17p) and/or TP53 mutation treated with acalabrutinib monotherapy or in combination with obinutuzumab for 48 months had significantly higher PFS rates compared with those treated with chlorambucil combined with obinutuzumab [45].

#### Treatment-naïve patients with CLL/SLL with or without IGHV mutations

Multiple studies have demonstrated the efficacy of BTK inhibitors over chemoimmunotherapy in patients with and without IGHV mutation. IGHV mutational status is considered to be one of the prognostic markers for disease progression with unmutated IGHV as an unfavorable prognostic marker [48]. Patients with unmutated IGHV genes are known to have worse outcomes following chemotherapy or chemoimmunotherapy [49]. However, the advent of BTK inhibitors has changed the course of action with improved responses in patients with IGHV-unmutated CLL [50].

The phase III ECOG-ACRIN E1912 study showed that ibrutinib in combination with rituximab showed better PFS than the FCR regimen in patients without IGHV mutation (HR: 0.26; 95% CI: 0.14–0.50) [41]. In the SEQUOIA study, a consistently longer PFS was observed with zanubrutinib monotherapy compared with BR regimen even in patients with unmutated IGHV [38]. Similarly, in the ELEVATE-TN study, acalabrutinib either as monotherapy or in combination with obinutuzumab showed effectiveness in patients with unmutated IGHV (48-month PFS rates: 77.1% in the acalabrutinib monotherapy group and 85.7%, in the acalabrutinib plus obinutuzumab group) [51].

Although BTK inhibitor therapy looks superior to chemoimmunotherapy in most patient populations in TN setting, conclusion cannot be drawn on the most effective frontline therapy in young and fit patients without del(17p) and/or TP53 mutation and with mutated IGHV because of the limited course of chemoimmunotherapy and the PFS plateau observed with long-term follow-ups.

#### Patients with R/R CLL/SLL

The advent of BTK inhibitors has revolutionized the treatment of patients with R/R CLL/SLL. Table 3 summarizes the major clinical studies on BTK inhibitors for R/R CLL/SLL. BTK inhibitors have shown better efficacy than chemoimmunotherapy in patients with R/R CLL [52]. A 6-year follow-up of the RESONATE study in patients with R/R CLL receiving ibrutinib showed efficacy irrespective of high‐risk clinical or genomic features with a tolerable safety profile [53]. Patients receiving ibrutinib showed a significantly longer PFS than those receiving ofatumumab (44.1 months vs. 8.1 months; HR: 0.148; 95% CI: 0.113–0.196; *P* < 0.001). No new safety concerns were observed with ibrutinib confirming the long-term efficacy and safety of ibrutinib in previously treated patients with CLL [53]. However, the head-to-head comparison of BTK inhibitors showed zanubrutinib to be superior to ibrutinib in efficacy (HR: 0.65; 95% CI: 0.49–0.86; *P* = 0.002). Moreover, the new-generation BTK inhibitors showed better safety profiles than ibrutinib especially in terms of cardiovascular events [54, 55].

#### Patients with R/R CLL/SLL with or without del(17p) and/or TP53 mutation

In patients with CLL/SLL, the del(17p) or TP53 gene mutation is the most important single poor prognostic factor [56]. Previously, patients with R/R CLL/SLL with or without del(17p) and/or TP53 mutation were heavily treated; however, they were associated with poor response to classical chemoimmunotherapy [57]. In the ASCEND study, R/R CLL patients with or without del(17P) and/or TP53 mutation treated with acalabrutinib monotherapy had a significantly longer PFS than those receiving investigator’s choice (idelalisib plus rituximab [IR] or BR) with an acceptable safety profile [52]. Two randomized studies in patients with R/R CLL provided a direct comparison of first- and new-generation BTK inhibitors (ELEVATE-RR: ibrutinib vs. acalabrutinib; ALPINE: ibrutinib vs. zanubrutinib) [54, 55]. In the head-to-head, phase III, ELEVATE‑RR study of acalabrutinib and ibrutinib, the median PFS in both the groups was 38.4 months (95% CI: 33.0–38.6 and 95% CI: 33.0–41.6 in acalabrutinib and ibrutinib, respectively) with no statistically significant difference observed between acalabrutinib and ibrutinib [54]. However, zanubrutinib demonstrated a statistically significant improvement in PFS when compared with ibrutinib in high-risk patients with del(17p) and/or TP53 mutation (72.6% vs. 54.6%, HR: 0.53; 95% CI: 0.31–0.88). At 24 months, the PFS rate was 78.4% in the zanubrutinib group and 65.9% in the ibrutinib group. Moreover, zanubrutinib showed a safety profile better than that of ibrutinib with fewer cardiac disorders compared with the ibrutinib group, and none of the patients in the zanubrutinib group had cardiac disorder–related death, whereas six patients who received ibrutinib had fatal cardiac disorders [55].

#### Patients with R/R CLL/SLL with or without IGHV mutation

IGHV mutated or unmutated patients with R/R CLL/SLL have benefited from BTK inhibitors. In a phase III, ASCEND study, better PFS benefit was observed with acalabrutinib monotherapy than IR or BR regimen in patients with unmutated IGHV (*P* < 0.001) and mutated IGHV (*P* = 0.027) [58]. Similarly, the RESONATE study demonstrated that the efficacy of ibrutinib was better than that of ofatumumab in both IGHV mutated (HR: 0.103; 95% CI: 0.067–0.159) and unmutated (HR: 0.200; 95% CI: 0.113–0.353) patients. The direct comparison of ibrutinib with zanubrutinib in the ALPINE study showed that the efficacy of zanubrutinib was superior to ibrutinib in IGHV unmutated patients (HR: 0.64; 95% CI: 0.47–0.87) [55].

##### Recommendations

- BTK inhibitors are recommended in patients with CLL/SLL regardless of age, fitness, del(17p) and/or TP53 mutation, IGHV mutational status, and therapeutic settings (frontline or salvage; category 1; Agree: 100%, 18/18).
- Despite no apparent impact on decision-making regarding BTK inhibitor utility, tests for common prognosis biomarkers, such as del(17p) and/or TP53 mutation and IGHV mutational status, are still recommended for both scientific interests and long-term therapeutic planning after failure of BTK inhibitor (category 2A; Agree: 94%, 17/18).
- Acalabrutinib and zanubrutinib have a more favorable safety profile than ibrutinib, especially in terms of cardiovascular events. Furthermore, zanubrutinib has also shown a superior efficacy profile than ibrutinib. Based on the results of head-head comparison studies, zanubrutinib could be recommended as the most preferred treatment regimen for patients with CLL/SLL (category 1; Agree: 94%, 17/18).

### Mantle cell lymphoma

Mantle cell lymphoma (MCL) is a distinct subtype of NHL with highly heterogenous presentation and aggressive clinical course [59, 60]. Although chemoimmunotherapy and stem cell transplantation have improved the outcome of patients with MCL, most of them had a relapse and are subjected to treatment-emergent AEs [61].

#### Patients with TN MCL

BTK inhibitors can be considered in previously untreated patients with MCL, if conventional chemotherapy cannot be tolerated. Table 4 presents the vital studies of BTK inhibitors in the treatment of patients with MCL. The indolent form of MCL requires individualized management. The IMCL-2015 study showed the efficacy of ibrutinib in combination with rituximab with a high rate of complete response (CR) and undetectable minimal residual disease (MRD) observed in indolent clinical forms of MCL [62]. BTK inhibitors are the preferred choice of treatment for patients with MCL considered to be unsuitable candidates for autologous stem cell transplantation (ASCT). The SHINE study involving elderly patients with previously untreated MCL evidenced the benefit of BTK inhibitors as a significantly prolonged PFS was observed with ibrutinib plus standard chemoimmunotherapy consisting of bendamustine at a dose of 90 mg/m^2^ of body surface area and rituximab at a dose of 375 mg/m^2^ (median PFS: 80.6 months in the ibrutinib group and 52.9 months in the placebo group) [63]. Several exploratory studies using BTK inhibitors in MCL are ongoing to evaluate the efficacy of BTK inhibitors in patients with MCL [64, 65]. The results from the ongoing BGB‑3111‑306 study (MANGROVE) in TN patients with MCL who are ineligible for ASCT comparing the efficacy of zanubrutinib plus rituximab with BR may provide further insight into the efficacy of BTK inhibitors for the treatment of patients with MCL [65].

**Table 4:** Evidence of BTK inhibitors in patients with MCL

Patients type	Treatment regimen	Efficacy	Study	Phase	Patients (N)
Patients with treatment naïve MCL					
Indolent clinical forms	Ibrutinib + rituximab	ORR: 84%; CR rate: 80%; estimated PFS at 36 months: 93%	IMCL-2015 [62]	2	50
Untreated MCL	Zanubrutinib + rituximab followed by rituximab (375 mg/m^2^), dexamethasone (20 mg), cytarabine (2000 mg/m^2^), and oxaliplatin (130 mg/m^2^) (R-DHAOx) then zanubrutinib maintenance	CR rate: 88.2% (15/17); MRD negative rate: 100% Study ongoing	NCT04624958 [173]	2	17
ASCT eligible	Ibrutinib + R-CHOP followed by w/wo ASCT + ibrutinib maintenance	ORR: 98%; CR rate: 45%; 3-year FFS rate: 86%	TRIANGLE [66]	3	807
	Zanubrutinib + rituximab 375 mg/m^2^, cyclophosphamide 750 mg/m^2^, doxorubicin 50 mg/m^2^, vincristine 1.4 mg/m^2^, prednisone 100 mg, (R-CHOP)/ dexamethasone 40 mg, rituximab 375 mg/m^2^, cytarabine 2 × 2 g/m^2^, cisplatin 100 mg/m^2^ (R-DHAOx) ± ASCT	CR: 85.7% (6/7); MRD negative rate: 100% Study ongoing	NCT04736914 [64]	2	47
ASCT ineligible	Ibrutinib + BR vs. placebo + BR	Median PFS: 80.6 vs. 52.9 months; CR rate: 65.5% vs. 65.5%	SHINE [63]	3	523
	Zanubrutinib + rituximab vs. BR	Ongoing	MANGROVE [65]	3	500
Patients with R/R MCL					
Overall	Zanubrutinib	ORR: 83.7%; CR rate: 77.9%; median PFS: 33 months	BGB-3111–206 [72]	2	86
	Acalabrutinib	ORR: 81%; CR rate: 40%; 12-month PFS rate: 67%, 12-month; OS rate: 87%	ACE-LY-004 [14]	2	124
	Ibrutinib	ORR: 68%; CR rate: 21%; median PFS: 13.9 months	PCYC-1104 [8]	2	111
	Orelabrutinib	ORR: 82.5%; CR rate: 24.7%	ICP-022-MCL [73]	2	97

ASCT, autologous stem cell transplantation; BR, bendamustine plus rituximab; BTK, Bruton tyrosine kinase; CR, complete response; FFS, failure-free survival; MCL, mantle cell lymphoma; MRD, minimal residual disease; PFS, progression-free survival; R-CHOP, rituximab, cyclophosphamide, doxorubicin, vincristine, and prednisone; R/R, relapsed/refractory; SLL, small lymphocytic lymphoma; ORR, overall response rate; OS, overall survival

Patients suitable for ASCT received induction with rituximab, cyclophosphamide, doxorubicin, vincristine, and prednisone (R-CHOP) or rituximab, dexamethasone, cytarabine, and cisplatin (R-DHAP) and were randomly assigned to the control arm (R-CHOP or R-DHAP induction followed by ASCT and observation), ASCT-ibrutinib arm (R-CHOP plus ibrutinib or R-DHAP induction followed by ASCT and 2 years of ibrutinib maintenance), and ibrutinib monotherapy arm (R-CHOP plus ibrutinib or R-DHAP and 2 years of ibrutinib maintenance) in the phase III, TRIANLGE study [66]. After a median follow-up of 31 months, the addition of ibrutinib during induction and as maintenance with or without ASCT evidenced the efficacy of BTK inhibitor [67].

#### Patients with R/R MCL

Before the advent of BTK inhibitors, patients with R/R MCL had a limited therapeutic option with generally poor outcomes [68, 69]. Both national and international guidelines recommend the use of BTK inhibitors for the treatment of patients with R/R MCL [70, 71]. Table 4 presents the details of vital studies on BTK inhibitors used for the treatment of patients with R/R MCL. A long-term follow-up of a phase II study showed durable responses and tolerable safety profile of zanubrutinib in patients with R/R MCL [72]. Similarly, acalabrutinib demonstrated clinically meaningful survival benefits and a favorable safety profile in the treatment of patients with R/R MCL [14]. Ibrutinib as a monotherapy in patients with R/R MCL also showed durable response and favorable toxicity profile [8]. Furthermore, Chinese patients with R/R MCL have demonstrated sustained efficacy and long-term safety with orelabrutinib in a phase II study [73]. In April 2023, ibrutinib was voluntarily withdrawn from the US market as the treatment option for patients with R/R MCL, but the decision has no impact on ibrutinib in China so far [74]. For the treatment of R/R MCL, initiating BTK inhibitors at the earliest can provide better therapeutic efficacy to the patients. A meta-analysis of zanubrutinib in R/R MCL showed that patients who received zanubrutinib as the second-line therapy was associated with better survival outcomes than those who received it as later-line therapy [75]. The ORR and CR rates of the zanubrutinib monotherapy for MCL are generally higher than those of the first-generation BTK inhibitor monotherapy. This may be due to the structural optimization of the new-generation BTK inhibitors, resulting in higher target occupancy and longer inhibition time. Recently, non-covalent BTK inhibitor, pirtobrutinib has demonstrated the effectiveness as monotherapy in patients with R/R MCL with an ORR of 58% (95% CI: 46.9–68.1) [76]; however, it has not yet been approved in China, Hong Kong, Taiwan, and Macau.

##### Recommendations

For TN patients

- BTK inhibitors combined with chemoimmunotherapy are recommended for the treatment of patients with MCL aged ≥ 65 years or frail patients (category 2B; agree: 83%, 15/18).
- BTK inhibitors are recommended for the treatment of patients both suitable and unsuitable for ASCT during induction and as a maintenance therapy (category 2B; Agree: 78%, 14/18).

For R/R patients

- BTK inhibitors are the preferred treatment of choice for patients with R/R MCL (category 2A) and are recommended to start treatment with BTK inhibitors as early as possible for better outcomes (category 2A; Agree: 94%, 17/18).
- Acalabrutinib and zanubrutinib have more favorable safety profiles than ibrutinib, especially in terms of cardiovascular events. Zanubrutinib further demonstrated a superior efficacy profile compared with ibrutinib. Based on the results of head-to-head comparison studies, zanubrutinib is recommended as the most preferred treatment regimen for patients with MCL (category 2B; Agree: 72%, 13/18).

### Diffuse large B-cell lymphoma

Diffuse Large B-Cell Lymphoma (DLBCL) is the most common aggressive NHL with an incidence of 7 cases per 100,000 people per year [77]. DLBCL can be divided into germinal center B cell (GCB), activated B cell (ABC), or non-GCB type and unclassified type by genotyping. Molecular classification of DLBCL helps to personalize the therapy for DLBCL [78].

Currently, there is no standard BTK inhibitor-based regimen for the treatment of patients with newly diagnosed DLBCL. In addition to BTK inhibitor monotherapy, the combination of BTK inhibitors and R-CHOP regimen is also used. The results of the PHOENIX study showed that in previously untreated patients with ABC DLBCL, ibrutinib in combination with R-CHOP regimen did not meet the primary endpoint of the study as the event-free survival (EFS) was not improved with the combination therapy [79]. Moreover, the addition of ibrutinib did not have significant difference in the PFS (70.8% vs. 68.1%), OS (82.8% vs. 81.4%), and CR (67.3% vs. 68.0%) rates. The ESCALADE study of acalabrutinib in combination with R-CHOP is currently ongoing in patients with TN non-GCB aged ≤ 65 years. The results of the study will provide evidence on the beneficial effects of acalabrutinib addition to R-CHOP regimen in patients aged ≤ 65 years with untreated non-GCB DLBCL [80]. Multiple studies have evidenced that patients with R/R DLBCL can be effectively treated with BTK inhibitors [70]. The latest CSCO guidelines added zanubrutinib, a new generation of BTK inhibitor for the treatment of patients with R/R DLBCL [71]. The evidence of studies is presented in Table 5.

**Table 5:** Evidence of BTK inhibitors in patients with DLBCL

Patients type	Treatment regimen	Efficacy	Study	Phase	N
Non-GCB					
TN	Ibrutinib + R-CHOP (rituximab 375 mg/m^2^, cyclophosphamide 750 mg/m^2^, doxorubicin 50 mg/m^2^, vincristine 1.4 mg/m^2^, and oral prednisone [or equivalent] 100 mg) vs. placebo + R-CHOP	36-month PFS rate: 70.8% vs. 68.1%; 36-month OS rate: 82.8% vs. 81.4%; CR rate: 67.3% v 68.0%	PHOENIX [79]	3	419 vs. 419
TN, ≤ 65 years	Acalabrutinib + R-CHOP	Ongoing	ESCALADE [80]	3	600
R/R	Zanubrutinib	ORR, non-GCB: 36%, GCB: 25%	BGB-3111–207 [83]	2	29
R/R, ASCT ineligible	Zanubrutinib + lenalidomide	Best ORR: 90.9%, CR: 36.4% Study ongoing	BGB-3111–110 [84]	1	27
R/R	Ibrutinib	ORR, non-GCB: 37%, GCB: 5%	NCT00849654 NCT01325701 [85]	2	80
R/R	Acalabrutinib	ORR, non-GCB: 24% (5/21); 4 CR and 1 PR	NCT02112526 [86]	1b	21
BCL2/MYC expression					
TN	Ibrutinib + R-CHOP	DE: CR: 67.5%; PR 22.8%	PHOENIX, post hoc [174]	3	200
R/R	Ibrutinib	DE: ORR 47%; CR 37%	Landsburg et al. [90]	Case-series	25
R/R	Zanubrutinib	DE: ORR 61%; NDE: ORR 29%	BGB-3111–207 [83]	Post-hoc	121
TN	Zanubrutinib + R-CHOP	Ongoing	NCT05189197 [92]	2	41
CD79B, MYD88 (MCD)					
TN	Ibrutinib + R-CHOP	MCD and aged ≤ 60 years: 3-year EFS and OS: 100%	PHOENIX, post hoc [94]	3	31
TN	Orelabrutinib + R-CHOP	Ongoing	NCT05234684 [95]	3	150
R/R	Zanubrutinib	CD79B mutation: ORR 46%; MYD88 mutation: ORR 40%; MYD88 + CD79B (MCD) mutation: ORR 50%	BGB-3111–207 [83]	2	41
Elderly and unfit/frail					
TN	Ibrutinib + rituximab + lenalidomide	ORR 66.7%; CR rate: 56.7%; 2-year PFS rate: 53·3%; 2-year OS rate: 66·7%	NCT03949062 [99]	2	30
TN	Zanubrutinib + rituximab + lenalidomide vs. R-mini-CHOP (rituximab 375 mg/m^2^ on day 1, cyclophosphamide 400 mg/m^2^, doxorubicin 25 mg/m^2^, and vincristine 1 mg on day 2, and prednisone 40 mg/m^2^ on days 2–6, every 21 days)	Ongoing	NCT05179733 [100]	3	280

ASCT, autologous stem cell transplantation; BCL2, B-cell lymphoma 2; BTK, Bruton tyrosine kinase; CR, complete response; DE, double expressor, DLBCL, diffuse large B-cell lymphoma; EFS, event-free survival; GCB, germinal center B cell; MYC, myelocytomatosis oncogene; NDE, non-double expressor; ORR, overall response rate; OS, overall survival; PFS, progression-free survival; R-CHOP, rituximab, cyclophosphamide, doxorubicin, vincristine, and prednisone; R/R, relapsed/refractory; TN, treatment naïve

#### Non-GCB/ABC DLBCL

B-cell receptor (BCR) signaling can be activated via two different pathways: antigen-dependent signaling and antigen-independent tonic signaling. In non-GCB DLBCL, the constitutive activation of BCR and NF-κB signaling was associated with lymphomagenesis and cancer cell survival, which may explain the relationship between BTK inhibitor treatment and the response of patients with non-GCB DLBCL [81, 82].

The BGB-3111-207 study showed the efficacy of zanubrutinib in patients with R/R DLBCL. Furthermore, patients with non-GCB showed better ORR than patients with GCB DLBCL (36% vs. 25%) [83]. Zanubrutinib in combination with lenalidomide has shown efficacy even in patients with R/R DLBCL who are not eligible for ASCT [84]. A phase I/II clinical trial in patients with R/R DLBCL showed better survival outcomes in those with ABC DLBCL than those with GCB DLBCL (ORR: 37% vs. 5%) supporting the use of the ibrutinib-based therapy in patients with ABC DLBCL [85]. Acalabrutinib monotherapy has also demonstrated efficacy in patients with non-GCB R/R DLBCL (ORR: 24%) [86].

#### Patients with BCL2/MYC expression, CD79B/MYD88 mutation

Patients with DLBCL having translocation of both myelocytomatosis oncogene (MYC) and B-cell lymphoma 2 (BCL2) are known to have an aggressive clinical course and poor outcome [87, 88]. In a post hoc subgroup analysis of TN patients in the phase III, PHOENIX trial, a numerical trend was observed toward improved EFS and PFS with ibrutinib in combination with R‐CHOP when compared with R-CHOP alone in patients with high MYC/BCL2 co‐expression [89]. Ibrutinib as monotherapy also demonstrated the efficacy with an ORR of 47% and a CR rate of 37% in patients with R/R non-CGB DLBCL with co-expression of MYC and BCL2 protein in a case series [90]. Zanubrutinib also showed beneficial effects in patients with R/R DLBCL with MYC/BCL2 co‐expression. Patients with MYC and BCL2 double-expressor DLBCL showed a higher ORR (61% vs. 29%), longer PFS (5.4 months vs. 3.6 months), and OS (10 months vs. 7 months) than non-double expressors [91]. Currently, a phase II study evaluating the efficacy of zanubrutinib plus R-CHOP is ongoing in patients with DLBCL with co-expression of BCL2 and MYC, which may provide the evidence on the efficacy of zanubrutinib addition in TN patients [92].

MYD88/CD79B (MCD) mutation is the most common mutation associated with ABC subtype DLBCL arising in immune-privileged sites, enriched with MYD88 L265P and/or CD79B gain-of-function mutations. MYD88 is a key molecule mediating Toll-like receptor signaling, whereas CD79B is part of the B-cell receptor complex that plays a role in maintaining the cell surface expression of the receptor [93].

The survival benefit of addition of ibrutinib to R-CHOP chemotherapy was observed in younger patients with MCD subtype of DLBCL. Patients who received ibrutinib in combination with R-CHOP showed a 3-year EFS and OS of 100%, compared with a significantly lower EFS and OS of 48% and 69.6%, respectively, with R-CHOP monotherapy [94]. An ongoing phase III BELIEVE-01 study of orelabrutinib plus R-CHOP in TN DLBCL with MCD will provide evidence on the efficacy of orelabrutinib in this patient population [95]. Zanubrutinib also showed efficacy in patients with non-GCB DLBCL and CD79B mutations, where patients with CD79B mutations showed a significantly higher ORR than those without CD79B mutations (60% vs. 26.9%; *P* = 0.005) [91].

#### Elderly and unfit/frail patients

The incidence of DLBCL increases with age, especially for those aged > 75 years [96]. Treatment of DLBCL in elderly patients poses a challenge due to high chances of remission failure associated with comorbidities and standard immunochemotherapy intolerance [97]. Thus, the reduced-intensity regimens (R-miniCHOP) are a useful option for treating elderly or unfit patients with DLBCL. However, the benefit of R-miniCHOP in unfit population remains uncertain as a relatively high proportion of drug discontinuation occurs due to toxicity [98].

Ibrutinib, rituximab, and lenalidomide in TN unfit or frail patients with DLBCL aged ≥ 75 years showed CR, ORR, 2-year PFS, and 2-year OS of 56.7%, 66.7%, 53.3%, and 66.7%, respectively, suggesting clinical effectiveness and safety of ibrutinib in combination with rituximab and lenalidomide as the first-line treatment in older patients with DLBCL [99]. An ongoing phase III study comparing the efficacy of zanubrutinib in combination with rituximab and lenalidomide with R-mini-CHOP will provide evidence on the efficacy of zanubrutinib in TN, unfit or frail elderly patients with DLBCL [100].

##### Recommendations

- BTK inhibitors are recommended as an optional treatment regimen in patients with non-GCB DLBCL (category 2B; Agree: 67%, 12/18) and in patients with DLBCL having specific subtypes (correlation of BCL2/MYC expression, MCD mutation; category 2A; Agree: 94%, 17/18).
- For patients with poor response or unfit (elderly or frail) for standard chemotherapy (i.e., R-CHOP), BTK inhibitors with less intensity chemotherapy (i.e., R-mini-CHOP) or chemo-free regimen (BTK inhibitors with rituximab and/or lenalidomide) could be recommended (category 2B; Agree: 83%, 15/18).

### Central nervous system lymphoma

Central nervous system lymphoma (CNSL) is an uncommon type of NHL that originates within the central nervous system (CNS). It is considered an extranodal lymphoma and primarily affects the brain, spinal cord, and leptomeninges [101]. The diagnosis and treatment of CNSL can be challenging due to the unique anatomical location of the tumor and the limited number of effective therapies. However, advancements in the understanding of the biology of CNSL and the development of novel treatment strategies have improved outcomes for patients with this disease [102]. In 2022, a Chinese expert consensus for the management of primary CNSL has been published, which suggested that patients with R/R primary CNSL can be treated with BTK inhibitors with or without high-dose chemotherapy as re-induction therapy [103]. The evidence of studies on BTK inhibitors in patients with CNSL is summarized in Table 6.

**Table 6:** Evidence of BTK inhibitors in patients with CNSL

Patients type	BTK inhibitor regimens	Efficacy	Study	Phase	N
TN, R/R CNSL	TN: zanubrutinib + rituximab R/R: zanubrutinib + HD-MTX (methotrexate at 3.5–5.0 g/m^2^ d1 and cytarabine at 2.0 g every 12 h on d2 and d3, every 21 days per cycle) + rituximab	TN: ORR CR 100% (5/5) R/R: ORR, CR 60% (3/5)	Zhang et al. [26]	Retrospective	10
	Orelabrutinib + immunotherapy + chemotherapy + radiotherapy	TN: ORR 100% (4/4); CR 25% (1/4) R/R: ORR 60% (9/15); CR 26.6% (4/15)	Wu et al. [104]	Retrospective	19
R/R CNSL	Tirabrutinib	ORR: 64%; CR/CRu 34%; PFS: 2.9 months; OS: NR	ONO-4059 [105]	2	44
	Ibrutinib	ORR: 59%; CR: 23%; PFS: 4.8 months; OS: 19.2 months	NCT02542514 [107]	2	52
	Zanubrutinib + HD-MTX + rituximab	Achieved CR after adding zanubrutinib for 3 cycles	Cheng et al. [110]	Case report	1
R/R CNSL/SCNSL	Ibrutinib + HD-MTX + rituximab	ORR: 80%; CR: 53.3%	NCT02315326 [108]	1b	15
R/R CNSL/PVRL	Ibrutinib + DA-TEDDi-R	ORR: 93%; CR: 86%	Lionakis et al. [25]	1b	16
R/R CNSL	Orelabrutinib + lenalidomide + rituximab + HD-MTX + TMZ	ORR: 86.7% (13/15); CR: 73.7% (11/13)	Yang et al. [109]	Retrospective	15

BTK, Bruton tyrosine kinase; CNSL, central nervous system lymphoma; SCNSL, secondary CNSL; CR, complete response; DA-TEDDi-R, rituximab, liposomal doxorubicin, temozolomide, etoposide and dexamethasone; HD-MTX, high-dose methotrexate; ORR, overall response rate; OS, overall survival; PFS, progression-free survival; PVRL, primary vitreoretinal lymphoma; R/R, relapsed/refractory; TN, treatment naïve

#### Patients with TN CNSL

A retrospective evaluation of primary CNSL revealed that all patients (100%) achieved a CR. Out of five patients, four patients were treated with zanubrutinib + rituximab whereas one patient was treated with only zanubrutinib [26]. In a retrospective study of patients receiving an orelabrutinib-based regimen, 4 patients with TN CNSL achieved an ORR of 100%, with one patient achieving CR rate of 25%. The study also found that both the 6-month PFS and OS rates were 100% [104].

#### Patients with R/R CNSL

In 2022, tirabrutinib (ONO-4059) was approved for the treatment of R/R CNSL in Taiwan. In a phase I/II study involving 44 patients with R/R primary CNSL treated with tirabrutinib, ORR was observed in 64% of patients, with 34% achieving CR/unconfirmed CR (CRu), which indicated favorable efficacy of tirabrutinib in patients with R/R primary CNSL [105]. Currently, an open-label, phase II PROSPECT study (NCT04947319) is evaluating the safety and efficacy of tirabrutinib for patients with newly diagnosed or R/R primary CNSL that may provide further insight into tirabrutinib for the treatment of patients with primary CNSL [106].

A retrospective study analyzed the outcomes of five patients with R/R CNSL who received zanubrutinib-containing regimens. Of these patients, 60% (3 out of 5) achieved CR [26]. Similarly, in another study consisting of patients with R/R CNSL who received an orelabrutinib-based regimen, 60% of patients achieved an ORR, with 26.6% (4 patients) achieving CR [104]. Several phase Ib/II studies have demonstrated the effectiveness of ibrutinib-based regimens for the treatment of CNSL. In a phase II study, ibrutinib monotherapy was shown to have a disease control rate of 70%, with 19% of patients achieving a CR. The median PFS and OS were 4.8 months (95% CI: 2.8–12.7) and 19.2 months (95% CI: 7.2–NR), respectively [107]. A phase Ib trail demonstrated 80% ORR when patients were treated with ibrutinib-based regimens. The median PFS for all patients was 9.2 months, and the 1-year OS rate was 71.1% [108]. In a retrospective study, patients with primary CNSL (PCNSL) received orelabrutinib-based regimens and showed that the ORR was 86.7%, with 73.3% of patients achieving CR. Additionally, the study found that circulating tumor DNA (ctDNA) levels in both blood and CSF were closely associated with tumor recurrence and treatment response [109]. A case report described a 53-year-old man with R/R PCNSL who was treated with zanubrutinib and achieved CR [110].

##### Recommendations

- It is recommended that patients with CNSL be treated with BTK inhibitor-based regimens, either alone or in combination with chemotherapy, as a treatment approach for induction/re-induction and the maintenance therapy in both TN and R/R patients (category 2B; Agree: 72%, 13/18).

### Waldenström macroglobulinemia

Waldenström macroglobulinemia (WM) is a type of rare lymphoproliferative disorder that is characterized by the abnormal production of monoclonal immunoglobulin M (IgM) protein and the infiltration of lymphoplasmacytic cells into the bone marrow. The B-cell receptor signaling pathway is an important factor in the development of WM, and, hence, BTK inhibitors are a promising therapeutic option for this disease [111]. Overall, BTK inhibitors have expanded the range of treatment options available for patients with WM, making them a well-tolerated and effective treatment option for both newly diagnosed and R/R cases. A phase III trial compared the efficacy of new generation BTK inhibitors, zanubrutinib with ibrutinib, and demonstrated the effectiveness of both for WM with a trend toward better response quality and less toxicity, particularly cardiovascular toxicity associated with zanubrutinib [112]. For acalabrutinib and orelabrutinib, a lack of data exist from head-to-head studies to compare the efficacy or safety profile with the first-generation BTK inhibitor ibrutinib. Further exploration is needed to demonstrate the benefit of acalabrutinib and orelabrutinib. The evidence of studies on BTK inhibitors in patients with WM is summarized in Table 7.

**Table 7:** Evidence of BTK inhibitors in patients with WM

Patients type	Treatment regimen	Efficacy	Study	Phase	N
Patients with WM (Overall)					
TN	Ibrutinib + rituximab	PFS: NR (rate 68%); RR: 76%, PR: 45%; VGPR: 29%; CR: 1%; TTNT: NR	iNNOVATE [113]	3	75
R/R	Ibrutinib	RR: 77%; VGPR: 29%; PR: 48%	iNNOVATE [114]	3	31
R/R	Ibrutinib	ORR: 90.5%, MRR: 79.4%, 5-year OS: 87%	NCT01614821 [115]	2	63
TN	Zanubrutinib	MR: 87.5%; VGPR: 33.3%; PR: 54.2%	BGB-3111-AU003 [175]	1/2	24
R/R	Zanubrutinib	MR: 79.6%; VGPR: 49%; PR: 28.6%	BGB-3111-AU003 [175]	1/2	49
TN	Zanubrutinib	MR: 64%; VGPR: 27%; PR: 36%	NCT04052854 [176]	2	11
R/R	Zanubrutinib	MR 90%; VGPR: 43%; PR: 33%	NCT04052854 [176]	2	30
R/R	Zanubrutinib	MR: 69.8%; VGPR: 32.6%	BGB-3111–210 [177]	2	44
TN	Zanubrutinib	MR: 21%; PR: 47%; VGPR: 26%	ASPEN [112]	3	19
R/R	Zanubrutinib	MR: 16%; PR: 49%; VGPR: 29%	ASPEN [112]	3	83
TN	Acalabrutinib	ORR: 93%; MR: 14%; PR: 71%; VGPR: 7%	NCT02180724 [178]	2	14
R/R	Acalabrutinib	ORR: 94%; MR: 15%; PR 47%; VGPR: 32%	NCT02180724 [178]	2	92
R/R	Orelabrutinib	MR: 80.9%; VGPR: 21.3%; PR: 59.6%	ICP-CL-00105 [179]	2	47
WM patients with MYD88 mutation					
MYD88^L265P^CXCR4^WT^	Ibrutinib	ORR: 100%; MRR: 91.2%	NCT01614821 [119]	2	34
R/R: 34; TN: 2	Acalabrutinib	ORR: 94%; MRR: 78%	NCT02180724 [178]	2	36
MYD88^L265P^CXCR4^WT^	Orelabrutinib	MRR: 84.6%	ICP-CL-00105 [179]	2	–
MYD88 mutation	Zanubrutinib vs. ibrutinib	CR + VGPR: 36.3% vs. 25.3%	ASPEN [180]	3	201
WM patients with MYD88 wild type					
MYD88^WT^CXCR4^WT^	Ibrutinib	ORR: 71.4%; MRR: 28.6%	NCT01614821 [119]	2	7
R/R: 13; TN: 1	Acalabrutinib	ORR: 79%; MRR: 57%	NCT02180724 [178]	2	14
MYD88^WT^CXCR4^WT^	Orelabrutinib	ORR: 25%	ICP-CL-00105 [179]	2	–
MYD88WT	Zanubrutinib	MRR: 65% (including 34% CR + VGPR)	ASPEN [120]	3	28
WM patients with CXCR4 mutation					
MYD88^Mut^CXCR4^Mut^	Ibrutinib	MRR: 68.2%; VGPR: 9.1%; median PFS: 38%	NCT01614821 [115]	2	2
CXCR4 mutation	Zanubrutinib vs. ibrutinib	VGPR: 21% vs. 10% MRR: 79% vs. 65%	ASPEN [120]	3	201

BTK, Bruton tyrosine kinase; CR, complete response; ORR, overall response rate; OS, overall survival; MR, minor response; MRR, major response rate; NR, not reached; PFS, progression-free survival; PR, partial response; R/R, relapsed/refractory; TN, treatment naïve; TTNT, time to next line of therapy; VGPR, very good partial response; WM, Waldenström macroglobulinemia

#### Overall patients

Based on the results of several clinical trials, BTK inhibitors have emerged as an effective and well-tolerated treatment option for WM. The iNNOVATE trial, a randomized, double-blind, placebo-controlled study, showed that ibrutinib plus rituximab significantly improved PFS compared with placebo plus rituximab, with a 54-month PFS rate of 68% versus 25%, respectively, in patients with TN WM. Higher response rates (RRs; 76% vs. 31%) were obtained in ibrutinib plus rituximab versus placebo plus rituximab [113]. Similarly, the effectiveness of ibrutinib monotherapy in rituximab-refractory patients was also observed (60 months of PFS: 40%, ORR: 87%, RR: 77%) [114]. A long-term follow-up of ibrutinib in previously treated patients with WM also demonstrated the efficacy of ibrutinib with an ORR of 90.5%, a major response rate (MRR) of 79.4%, and a 5-year OS rate of 87% for all patients [115]. In the ASPEN trial, zanubrutinib demonstrated non-inferiority to ibrutinib with a very good partial response (VGPR) in both TN (26% vs. 17%) and R/R patients (29% vs. 20%) with WM. As zanubrutinib has a higher degree of selectivity, the safety profile also showed differences. Atrial fibrillation and hypertension were reported at greater frequencies with ibrutinib compared with zanubrutinib. As atrial fibrillation is a well-recognized complication of ibrutinib therapy and is relative to an age-matched controlled population, patients appear to be at a continuously increased risk for the development of atrial fibrillation over the course of therapy [112]. Additionally, a multicenter, phase II trial evaluated the activity and safety of acalabrutinib as a single agent and reported an ORR of 93% in both TN and R/R patients with 7% and 33% of TN and R/R patients, respectively achieving a VGPR [116]. These findings suggest that BTK inhibitors, including ibrutinib, zanubrutinib, and acalabrutinib, are highly effective and well-tolerated options for the treatment of patients with WM. So far, only phase II studies have evaluated the efficacy of acalabrutinib in patients with WM; hence, conclusion cannot be drawn on its efficacy in this patient population. Several phase III studies of acalabrutinib in patients with WM are warranted.

#### Patients with WM having MYD88 mutation

Activating somatic mutation of myeloid differentiation factor 88 (MYD88) is common and well investigated in WM. Mutation of MYD88 might lead to a BTK-mediated activation of NFκB resulting in nuclear translocation and malignant cell growth [117]. The presence of such mutations affects the prognosis and response to targeted therapies, in particular, BTK inhibitors [118].

Patients with R/R WM having MYD88 mutations with wild-type CXCR4 have shown better outcomes associated with ibrutinib monotherapy (ORR: 100%; MRR: 91.2%) [119]. In a phase II trial, out of 36 patients with MYD88^L265P^ mutation, the overall response and major response were reported in 34 (94%) and 28 (78%) patients, respectively, with acalabrutinib monotherapy [116]. The ASPEN, the largest phase III trial with a head-to-head comparison of zanubrutinib and ibrutinib showed higher CR + VGPR rate associated with zanubrutinib than ibrutinib (36.3% vs. 25.3%), demonstrating a long-term safety and better tolerability [120].

#### Patients with WM having wild-type MYD88

The prognosis of wild-type MYD88 (MYD88^WT^) tumors is poor [121]. Patients with MYD88^WT^ are known to have a lower response rate (none > 50%) to ibrutinib [122]. In contrast to the favorable outcomes with ibrutinib in patients with MYD88 mutation, the results for ibrutinib-treated patients with MYD88^WT^ tumors were poor (ORR: 71.4%; MRR: 28.6% for patients with MYD88^WT^ and CXCR4^WT^) [119]. In patients with MYD88^WT^ WM, treatment with orelabrutinib showed an ORR of only 25%, suggesting a poor response even with orelabrutinib in this patient population. Patients with MYD88^WT^ are less likely to benefit from BTK inhibitors than the mutation counterparts. A phase II trial reported an ORR and MRR of 79% and 57%, respectively, with acalabrutinib, which is much lower than those with MYD88 mutation subtypes [116]. Patients with MYD88^WT^ in the ASPEN trial who received zanubrutinib 160 mg twice a day demonstrated a CR with a MRR of 65% (including 34% CR + VGPR), suggesting that zanubrutinib can achieve a high response rate even in patients with MYD88^WT^ [120].

#### Patients with WM having CXCR4 mutation

Somatic mutations in the C-terminal domain of CXCR4 lead to CXCR4 signaling and are present in 30% to 35% of patients with WM [119]. CXCR4 mutation mostly occur in those with MYD88 mutations but some patients with MYD88^WT^ also express CXCR4 mutations [123]. Patients with WM having CXCR4 mutation is usually associated with a delayed response, fewer major responses, and shorter PFS to BTK inhibitors, particularly ibrutinib [115]. In a head-to-head comparison of zanubrutinib and ibrutinib in the ASPEN trial, zanubrutinib demonstrated better VGPR (21% vs. 10%) and MRR (79% vs. 65%) than ibrutinib in patients with CXCR4 mutation. Similar efficacy in VGPR (45% vs. 31%) was observed in patients with CXCR4^WT^ mutation [120].

##### Recommendations

- BTK inhibitors as a monotherapy or in combination with rituximab are recommended for the treatment of WM (category 1; Agree: 100%, 18/18).
- Zanubrutinib is one of the treatment options for patients with MYD88WT (category 2A; Agree: 100%, 18/18).
- Zanubrutinib is the preferred treatment option for patients with CXCR4 mutation (category 1; Agree: 89%, 16/18).
- Zanubrutinib is recommended as the preferred treatment regimen rather than ibrutinib considering the balance of efficacy and safety, especially for CV events in a head-to-head study (category 1; Agree: 100%, 18/18).

### Marginal zone B-cell lymphoma

Marginal zone B-cell lymphoma (MZL) is a subtype of NHL that originates from memory B cells in the marginal zone of lymphoid tissues [124]. It accounts for about 7% of all NHL cases and can affect different organs such as the spleen, lymph nodes, and mucosa-associated lymphoid tissue (MALT). Standard treatments for MZL include chemotherapy, immunotherapy, and radiation therapy, but R/R MZL remains challenging to treat, and there is a need for new treatment options [125]. The evidence of studies on BTK inhibitors in patients with MZL is summarized in Table 8.

**Table 8:** Evidence of BTK inhibitors in patients with MZL

Patients type	BTK inhibitor regimens	Efficacy	Study	Phase	N
Patients with R/R MZL	Zanubrutinib	ORR: 68.2%; CR rate: 25.8%; median PFS: NR	MAGNOLIA [126]	2	68
	Acalabrutinib	ORR: 53%; CR rate: 13%; median PFS: 27.4 months	ACE-LY-003 [127]	1/2	43
	Ibrutinib	ORR: 58%; CR rate: 10%; median PFS: 15.7 months	PCYC-1121 [128]	2	63

BTK, Bruton tyrosine kinase; CR, complete response; R/R, relapsed/refractory; MZL, marginal zone B-cell lymphoma; NR, not reached; ORR, overall response rate; OS, overall survival; PFS, progression-free survival; R/R, relapsed/refractory

#### Patients with R/R MZL

The clinical trials, namely MAGNOLIA, ACE-LY-003, and PCYC-1121, have investigated the efficacy of BTK inhibitors in patients with R/R MZL. The MAGNOLIA trial found that the single agent zanubrutinib resulted in a high ORR of 68.2%, with a CR of 25.8 [126]. The ACE-LY-003 trial demonstrated efficacy of acalabrutinib where patients had a median PFS of 27.4 months with achieving 67% of PFS rate at 12 months [127]. Finally, the PCYC-1121 trial showed that single agent ibrutinib had an ORR of 58%, with a median PFS of 15.7 months (95% CI: 12.2–30.4) with better outcomes in patients previously treated with rituximab (ORR: 81%; median PFS: 30.4 months) [128]. These results suggest that BTK inhibitors may be an effective treatment for R/R MZL, and further studies are needed to determine the optimal use of these drugs. In April 2023, ibrutinib was voluntarily withdrawn from the US market as the treatment option for patients with MZL who require systemic therapy and have received at least 1 prior anti-CD20-based therapy [74]. Till date, only zanubrutinib has been approved by the FDA for the treatment of patients with R/R MZL [129, 130].

##### Recommendations

- BTK inhibitor is recommended as one of the treatment options for patients with R/R MZL (category 2A; Agree: 94%, 17/18).
- Zanubrutinib is highly recommended considering a better safety profile than ibrutinib, especially in terms of cardiovascular events (category 1; Agree: 89%, 16/18).

### Follicular lymphoma

Follicular lymphoma (FL) is the second most common type of indolent NHL [131]. In patients with previously untreated FL, ibrutinib in combination with once-weekly rituximab for 4 weeks demonstrated clinical activity and durable responses with tolerable safety profile. At a median study follow‐up of 34 months in the combination therapy, the ORR was 85% in the first-line FL treatment [132]. An international phase III study (PERSPECTIVE) evaluating the efficacy of ibrutinib plus rituximab versus rituximab plus placebo in TN elderly and/or unfit patients with FL is ongoing [133].

However, unlike other B-cell malignancies, the studies supporting the use of BTK inhibitors for R/R FL is insubstantial, especially as a monotherapy. A phase II study that assessed the efficacy and safety of ibrutinib monotherapy in patients with R/R FL (N = 110) showed ORR of only 20.9%, failing to meet the primary efficacy endpoint for the study [134]. In a phase II ROSEWOOD study, zanubrutinib plus obinutuzumab demonstrated superior PFS over obinutuzumab monotherapy (68.3% vs. 45.8%) in patients with R/R FL, suggesting a beneficial effect associated with BTK inhibitors when given as a combination therapy [135].

#### Recommendations

- Zanubrutinib with obinutuzumab is recommended as one of the treatment options for patients with R/R FL (category 2B; Agree: 83%, 15/18).

## Clinical applications of BTK inhibitors and management of AEs

Different lymphoid malignancies have been extensively treated using BTK inhibitors owing to their positive clinical response, improved efficacy, and the ease of administration. However, there are some important factors that need further consideration before starting patients on BTK inhibitor therapy. The presence of comorbidities in patients including bleeding diathesis, the management of surgical procedures as well as incidence of AEs (hypertension and other cardiovascular diseases, liver and kidney dysfunctions) need to be carefully evaluated. Drug–drug and drug–food interactions as well as the presence of infection and vaccination in the patients need to be considered before starting them on BTK inhibitor therapy. It is also necessary to assess the presence of autoimmune diseases in patients before starting BTK inhibitor therapy, because the incidence of autoimmune complications is very common in lymphoid malignancies [136].

### Drug–drug interactions

BTK inhibitors have a range of drug–drug interactions with agents that are mainly metabolized by cytochrome P450 3A (CYP3A) pathway; hence, the co-administration of CYP3A inhibitors or inducers with BTK inhibitors should be used with caution. From the information listed in package inserts, the co-administration of ibrutinib, acalabrutinib, and zanubrutinib with a strong or moderate CYP3A inhibitor may increase the plasma concentration of BTK inhibitors. It is recommended to interrupt BTK inhibitor treatment when taking strong CYP3A inhibitors for a short term (such as anti-infectives for ≤ 7 days), and in case of moderate CYP3A inhibitors, patients need to be closely monitored for adverse reactions.

It is recommended to make dietary adjustments in patients who consume grapefruit juice and other foods that interact with the CYP3A enzyme system and might interfere with BTK inhibitor therapy. Thus, a collaborative monitoring of treatment course by the pharmacist and the medical team is required for optimal results during the BTK inhibitor treatment [137].

Acalabrutinib is absorbed less efficiently in patients receiving gastric acid–reducing agents leading to a decrease in the plasma concentration of acalabrutinib [138]. Therefore, it is recommended to avoid a concomitant use of proton pump inhibitors and acalabrutinib [23]. In addition, when using an H_2_-receptor antagonist (H2RA), acalabrutinib should be taken 2 h before (or 10 h after) receiving H2RA, with an interval of 2 h between antacids and acalabrutinib consumption is suggested [139]. It is notable that many patients with B-cell malignancies remain on gastric acid—reducing agents for a prolonged period of time; hence, there is a possibility of clinically relevant interaction between BTK inhibitors and gastric acid—reducing regimens [20].

### Investigation of genetic mutations

For successful treatment of patients with B-cell malignancies, the detection of the mutated or abnormal molecules or genes is necessary to guide the individualized treatment. Gene mutation detection plays an important role in the classification of hematologic tumors and the determination of the etiology and pathogenesis of hematologic diseases. Patients with B-cell malignancies are recommended to undergo genetic examinations for following mutations before the initiation of treatment or in cases of poor prognosis: (1) MCL: detection of TP53 mutation; (2) CLL/SLL: detection of *del(11q), del(17p)/TP53* deletion or mutation and IGHV mutation status, and so on; (3) WM: detection of MYD88 L265P and CXCR4 WHIM mutation; (4) DLBCL: detection of MYD88 L265P, CD79b, bcl-6, bcl-2, Notch1/2 and myc mutations [140].

### Safety and AEs management

Although BTK inhibitors are generally safe and well tolerated, several studies have shown incidences of unique toxicities that require monitoring for their optimal management in order to achieve the best possible outcomes in patients being treated with BTK inhibitors [141, 142]. Majority of the AEs were of grades 1 to 2. As the incidence of ≥ grade 3 was very low, which could be managed by prolonging the treatment time, the rate of patients discontinuing BTK inhibitor treatment due to AEs was very low. The first-generation BTK inhibitor ibrutinib showed off-target effects due to low specificity. The most common reason for discontinuing ibrutinib involved the incidence of AEs such as atrial fibrillation, bleeding events, arthralgias, rash, diarrhea, and cytopenia with discontinuation or dosage reduction in 12% of patients.

As compared with ibrutinib, new-generation covalent BTK inhibitors such as acalabrutinib and zanubrutinib are more selective in nature, with less off-target activity and better tolerability with lesser AEs. Discontinuation rates were only 9%–11% in patients treated with acalabrutinib. In the ALPINE study, the discontinuation rate due to AEs in the zanubrutinib arm was 7.8%, whereas it was 13% in the ibrutinib arm. The most common AEs after treatment with new-generation covalent BTK inhibitors are neutropenia, thrombocytopenia, rash, bruises, leukopenia, and so on, which can be managed according to the instructions and related guidelines [35, 45]. Headache is the most frequently occurring AE linked with acalabrutinib. Several phase III studies have shown that patients treated with ibrutinib had a higher incidence of atrial fibrillation than those treated with zanubrutinib or acalabrutinib [54, 112]. Neutropenia was found to be the most common AE in patients treated with zanubrutinib, but its occurrence did not lead to a significant increase in infection [34, 143]. The primary AEs associated with BTK inhibitors are included in the following section.

#### Hemorrhage

Hemorrhage is a commonly occurring AE in patients treated with ibrutinib, whereas its incidence is relatively less in patients treated with the new-generation BTK inhibitors. Grade 3 or higher bleeding events were observed in 2.3% and 1.3% of patients with MCL treated with zanubrutinib and acalabrutinib, respectively, whereas ibrutinib showed a slightly higher incidence rate of 6% [14, 72, 144]. Patients with grade 3 or higher hemorrhage should be permanently discontinued from BTK inhibitors unless the disorder is curable and the risk of rebleeding is acceptable. The increased risk of hemorrhage upon administration of BTK inhibitors may be due to impairment in collagen-induced platelet activation, similar to the effects of aspirin. In ibrutinib-treated and acalabrutinib-treated patients, BTK and TEC kinases are both irreversibly inhibited, and, hence, both are at equal risks of bleeding events [145, 146]. In the ASPEN study, the incidence of grade 3 or higher hemorrhage was 8.9% in the zanubrutinib arm and 10.2% in the ibrutinib arm in patients with WM.

Studies have suggested that co-administration of BTK inhibitors with direct oral anticoagulants, such as rivaroxaban, dabigatran etexilate, and apixaban, and antiplatelet agents may increase the risk of hemorrhage [147]. The assessment of effect of anti-coagulant or antiplatelet agent in addition to ibrutinib in the PCYC-1102 study recorded a major bleeding event in 9% and 4% of patients, respectively [148]. Therefore, a case-to-case risk-versus-benefit profile should be considered while co-administering BTK inhibitor with antiplatelet or anticoagulant therapy with close monitoring of any hemorrhage events. In addition, it is recommended to withhold BTK inhibitor administration for 3–7 days before and after surgery, depending on the type of surgery and the potential risk of a bleeding event.

#### Thrombocytopenia and neutropenia

Thrombocytopenia and neutropenia were commonly observed in patients with CLL on BTK inhibitor treatment [149]. In the ALPINE study, patients with R/R CLL showed the incidence of grade 3 or higher in 3.4% of patients in the zanubrutinib arm and 5.2% in the ibrutinib arm. Among the patients, 21% in the zanubrutinib arm and 18.2% in the ibrutinib arm experienced grade 3 or higher neutropenia, which may be due to on-target toxicity [34, 143]. Dose interruptions are recommended for first to third occurrences of grade 3 or 4 neutropenia and thrombocytopenia, and dose discontinuation is recommended after the fourth occurrence. The occurrences of neutropenia and thrombocytopenia are caused by various complex mechanisms of immune dysregulation that are a consequence of CLL disease [9].

#### Infection

Infections were common in patients treated with BTK inhibitors because patients with B-cell malignancy are immunocompromised and at a highly increased risk of infections despite receiving effective therapy. The incidence of infection (of any grade) is observed in > 50% of patients receiving BTK inhibitor treatment [9]. However, most of these infections are grade 1 or 2 and are easily managed without any dose adjustment. In the ALPINE study, the zanubrutinib arm showed 26.5% of grade 3 or higher infections in patients with CLL, whereas the ibrutinib arm showed a slightly higher incidence of 28.1% [150]. Similarly, in the ASPEN study, the zanubrutinib arm demonstrated a 21.8% incidence of grade 3 or higher infections in patients with WM, whereas the ibrutinib arm showed a higher incidence of 27.6% [112]. In the ACE-CL-006 study, acalabrutinib and ibrutinib-treated patients with CLL had comparable incidence rates of grade 3 or higher infections at 30.8% and 30.0%, respectively [54].

The prevalence of opportunistic infections further increases in patients with grade 3 or higher infection; the risk of opportunistic infections such as *Aspergillus fumigatus, Pneumocystis jirovecii*, and other infections is increased. In case of fever and other symptoms related to infection, the etiology and pathogenic microorganisms should be determined with the help of complete medical examination. Prophylactic treatment should be considered in case of high-risk patients, and these patients should be continuously monitored for infection and treated immediately [136, 151].

#### Hypertension

Hypertension is a common AE observed in patients treated with BTK inhibitors. In a study, among 562 patients with lymphoid malignancies receiving ibrutinib treatment, 78.3% of patients developed new or worsened hypertension, of which 17.7% were of 3 grade or higher, thereby suggesting ibrutinib treatment to be associated with a substantial increase in the incidence and severity of hypertension [152]. Furthermore, the ACE-CL-001 phase II study reported the long-term follow-up (41 months) results in which 18% patients had hypertension (all grades), 10% of which were of grades 1–2, and 7% were of grade 3 or higher [17]. Thus, the incidence of hypertension in patients treated with ibrutinib was significantly higher as compared with those treated with acalabrutinib. In the ASPEN study, the incidence of any grade and grade 3 or higher hypertension in patients treated with zanubrutinib was significantly lower than those treated with ibrutinib (14.9% vs. 25.5% and 9.9% vs. 24.4%) [112]. Similarly, in the ACE-CL-006 study, the incidence of hypertension in the acalabrutinib arm was lower than that in the ibrutinib arm (8.6% vs. 20.2% and 4.1% vs. 8.7%) [54].

The mechanism responsible for hypertension is proposed to be PI3k/Akt inhibition, thereby downregulating PI3K p110-alpha as well as nitrous oxide synthesis [152, 153]. Patients should be monitored for treatment-emergent hypertension and managed by judicious optimization of baseline hypertension before treatment initiation, require regular monitoring of blood pressure during clinic visits, and need appropriate medical therapy for hypertension. Antihypertensive medications may require dose modification following discontinuation of BTK inhibitor therapy [9, 154].

#### Atrial fibrillation

Atrial fibrillation has been reported in 6%–10% of untreated patients with CLL, which has been observed to increase with patients’ age [155, 156]. Clinical studies in patients under ibrutinib treatment showed the 2-year incidence rate between 10 and 16% [157]. Unlike first-generation BTK inhibitors, the incidence of atrial fibrillation is negligible in patients treated with the new-generation BTK inhibitors. When patients with WM treated with zanubrutinib and ibrutinib were compared for the safety results in the ASPEN study, the incidence of atrial fibrillation was 2.0% and 15.3%, respectively [112]. Another clinical study, the ALPINE study conducted in patients with CLL/SLL evaluated atrial fibrillation as the secondary endpoint. The results showed that the incidence of atrial fibrillation was 5.2% and 13.3% in patients treated with zanubrutinib and ibrutinib, respectively, thereby underlining a significantly lower atrial fibrillation in the zanubrutinib group [150]. In the ACE-CL-006 study, the incidence of atrial fibrillation was 9.4% in the acalabrutinib arm and 16% in the ibrutinib arm. These results from head-to-head comparison studies demonstrated the superiority of new-generation BTK inhibitors in terms of safety, especially in CV events [54].

Although the mechanism of BTK inhibitors related to atrial fibrillation is still unclear, it is thought that the inhibition of PI3K signaling, which is crucial for cardiac protection under stress that is regulated by BTK and TEC, may play a role in the incidence of atrial fibrillation [153].

Patients with cardiac risk factors, hypertension, and acute infection may have an increased risk of arrhythmia [158], so such patients should be treated with zanubrutinib to reduce the chances of atrial fibrillation. Patients should be monitored regularly for arrhythmias during treatment, and ibrutinib therapy should be withheld in patients with symptoms and/or signs of ventricular tachycardia. Patients presenting with symptoms of arrhythmia (such as palpitations, dizziness, fainting, chest discomfort, or new-onset dyspnea) should be clinically evaluated and should undergo an electrocardiogram as indicated. When atrial fibrillation occurs, the treatment should be adjusted in time. For patients with atrial fibrillation having symptoms that cannot be completely controlled, the drug can be restarted at the initial dose or half the dose after atrial fibrillation is fully controlled, according to the physician’s assessment.

#### Headache

Headache is one of the most frequently occurring AEs observed in 22%–51% of patients treated with acalabrutinib therapy [17, 45, 159]. The incidence of acalabrutinib treatment-related headaches is usually observed during the initial phase of treatment, which is mild in nature and occurs for a limited duration [160]. In pivotal studies, about 70% of patients showed grade 1 or 2 headaches, during initial cycles (particularly weeks 1–3) [17, 45]. In the ACE-CL-006 study, the incidence of any grade and grade 3 or higher headaches was significantly higher in the acalabrutinib arm than in the ibrutinib arm, at 34.6% versus 20.2% and 1.5% versus 0%, respectively [54]. These headaches can be managed mostly without any medical interventions, or can be effectively treated using acetaminophen or caffeine, while avoiding the use of nonsteroidal anti-inflammatory drugs, if possible. Only 1% of headaches lead to treatment discontinuation [159]. Although mechanism(s) for these headaches is still unclear, it could be caused by calcitonin gene-related peptide (CGRP) agonism, which is of interest, given the new class of migraine medications designed to work by antagonizing CGRP [161].

### BTK inhibitor intolerance and resistance

Although treatment with first- and new-generation BTK inhibitors has extensively showed positive clinical response in patients with B-cell malignancies, patients have developed primary and secondary resistance due to drug intolerance leading to treatment discontinuation [162, 163]. The preliminary results of the single-arm, open-label, multicenter, phase II BGB‑3111‑215 study conducted in patients with R/R B-cell malignancies (CLL/SLL, MCL, MZL, or WM; N = 64) who were intolerant to previous BTK inhibitors (ibrutinib and acalabrutinib) showed good efficacy and tolerability to treatment with zanubrutinib. There was no recurrence of AEs in 75% of patients with ibrutinib and acalabrutinib intolerance after receiving zanubrutinib treatment. After zanubrutinib treatment, the incidence of lower grade AEs was observed in 90% and 33% of patients with ibrutinib and acalabrutinib intolerance, respectively. All grade 4 intolerance events and 68.3% of grade 3 intolerance events did not recur after zanubrutinib treatment [164, 165].

In another phase II clinical study, 60 patients with CLL having ibrutinib tolerance were treated with acalabrutinib and tolerance levels of patients during acalabrutinib and ibrutinib treatments were compared. About 40% of patients reported similar AEs in both treatments, whereas 67% of AEs were of lower grade with acalabrutinib than ibrutinib treatment. Further, 57% of AEs observed with ibrutinib treatment did not recur upon treatment with acalabrutinib [166]. Multiple mechanisms responsible for the resistance of BTK inhibitors include mutations in BTK and downstream signaling molecules, such as PLCγ2, CARD11, and BCL10 leading to prolonged and BTK-independent NF-κB activation resulting in tumor cell growth. Further, overactivation of PI3K/Akt/mTOR and the non-canonical NF-κB pathways can also inhibit cancer cell apoptosis. Additionally, overexpression of integrin β1 in the microenvironment in association with activated PI3K pathway also facilitate tumor growth. All these mechanisms cumulatively contribute to the resistance against BTK inhibitors [5].

## Future perspectives

The emergence of resistance and toxicity are the major limitations that lead to treatment discontinuation. To improve survival outcomes in patient with BTK inhibitor intolerance, several approaches are being explored. The use of non-covalent reversible BTK inhibitors is currently being investigated. Some representative examples of these third-generation reversible BTK inhibitors include pirtobrutinib (LOXO-305), nemtabrutinib, vecabrutinib, and HMPL-760, which are effective against wild-type and mutant Cys481 malignancies [5]. In a phase I/II BRUIN trial, 276 patients with R/R CLL/SLL received pirtobrutinib and 75% of the patients discontinued prior to BTK inhibitor therapy due to resistance; the ORR was 74%, whereas the median PFS was 19.4 months. Nemtabrutinib also showed similar trend in the BELLWAVE-001 trial, with ORR of 56% and median PFS of 26.3 months among responders, despite a smaller sample size including 57 patients. Additionally, reversible BTK inhibitors such as HBW-3‑10 and HBW-3‑20 are still under development in China. Targeted therapies are being used for preventing the activation of bypassing signaling pathways, such as PI3K inhibitors, BCL-2 inhibitors, SYK and LYN inhibitors, and HSP90 inhibitors [167].

Moreover, combination of existing treatment options might also be a novel treatment option for B-cell malignancies. In the CAPTIVATE-FD, CAPTIVATE-MRD, and GLOW studies, the combination of ibrutinib plus venetoclax was adopted in a fixed treatment duration, MRD patients, and elderly or patients with comorbidities, respectively. In CAPTIVATE-FD, ORR was 97% and CR was 56%. However, in the CAPTIVATE-MRD study, the 3-year DFS was similar in the placebo arm and the ibrutinib combination arm. The efficacy and safety from these trials showed that such all-oral, chemotherapy-free regimen can provide a synergistic and comprehensive disease control.

Recently, the progress in the development of BTK-targeted proteolysis targeting chimeras (PROTACs) has gained interest as an effective and alternate strategy to inhibit BTK activity. PROTACs are structurally composed of three elements, one end is a warhead ligand that binds to the target protein, the other end is a ligand that binds to the E3 ubiquitin ligase, and a linker that couples the two ligands [168]. A preclinical study with UBX-382 exhibited outstanding degradation potency against various mutant BTKs, suggesting the use in patients with recurrent cancers, specifically ABC DLBCL, who have received prior treatment with BTK inhibitors [169]. Four BTK degraders, such as NX-2127 (NCT04830137) and NX-5948 (NCT05131022) from Nurix Therapeutics, HSK-29116 (NCT04861779) and BGB-16673 (NCT05006716) small molecule drugs from Haisco and BeiGene, respectively, have entered clinical trials. PROTACs have great therapeutic potential with their unique advantages, and with the development of technology and in-depth research, PROTACs are expected to provide clinical therapeutic benefits in the near future.

## Conclusion

BTK inhibitors such as ibrutinib, zanubrutinib, orelabrutinib, and acalabrutinib, have shown good clinical efficacy in the treatment of various B-cell malignancies with better safety profiles compared with traditional chemotherapy and chemoimmunotherapy regimens, especially for elderly patients or patients who cannot tolerate conventional chemotherapy. The comparison of different BTK inhibitors has gained interest in the recent years. The head-to-head comparison of BTK inhibitors in the ALPINE and ASPEN studies provided guidance for the optimal selection of BTK inhibitors to a certain extent. More studies such as head-to-head comparisons are warranted to guide on the optimal treatment option. The outcomes of patients who progress following BTK inhibitors, especially those with primary resistant disease, are dismal [170, 171]. There are several therapies in trials that will provide valuable information in the future. Patients that are intolerant to the first-generation BTK inhibitors may switch to new-generation BTK inhibitors to improve efficacy and safety. Multiple studies on new BTK inhibitors are ongoing, which may bring more therapeutic options for patients with B-cell malignancies. PROTACs are likely to overcome the resistance and toxicity associated with BTK inhibitors, and future research on the use of PROTACs may provide some evidence for patients with B-cell malignancies. Further progress on the research to provide more therapeutic options are anticipated and the breakthrough progress will be updated.

### Supplementary Information

**Additional file 1. Table S1**. Recommendations questionnaire

## Data Availability

All information in this consensus can be found in the additional files and references.

## Abbreviations

ABC: Activated B cell
AE: Adverse event
ASCT: Autologous stem-cell transplantation
ATP: Adenosine triphosphate
AUC: Area under the curve
BBB: Blood–brain barrier
BCL-2: B-cell lymphoma 2
BCR: B-cell receptor
BR: Bendamustine plus rituximab
BTK: Bruton tyrosine kinase
CGRP: Calcitonin gene-related peptide
CLL: Chronic lymphocytic leukemia
CNS: Central nervous system
CNSL: Central nervous system lymphoma
CR: Complete response
CRu: Unconfirmed CR
CSCO: Chinese Society of Clinical Oncology
CSF: Cerebrospinal fluid
ctDNA: Circulating tumor DNA
CYP3A: Cytochrome P450 3A
DLBCL: Diffuse large B-cell lymphoma
EFS: Event-free survival
FCR: Rituximab, fludarabine, and cyclophosphamide
FDA: Food and Drug Administration
FL: Follicular lymphoma
GCB: Germinal center B cell
H2RA: H_2_-receptor antagonist
HL: Hodgkin lymphoma
HR: Hazard ratio
IC_50_: Enzymatic half-maximal inhibitory concentration
IGHV: And immunoglobulin heavy-chain variable-region gene
IgM: Immunoglobulin M
MALT: Mucosa-associated lymphoid tissue
MCL: Mantle cell lymphoma
MRD: Minimal residual disease
MYC: Myelocytomatosis oncogene
MZL: Marginal zone B-cell lymphoma
NCCN: National Comprehensive Cancer Network
NHL: Non-Hodgkin lymphoma
ORR: Overall response rate
OS: Overall survival
PFS: Progression-free survival
PROTACs: BTK-targeted proteolysis targeting chimeras
R-CHOP: Rituximab, cyclophosphamide, doxorubicin, vincristine, and prednisone
R-DHAP: Rituximab, dexamethasone, cytarabine, and cisplatin
R/R: Relapsed/refractory
RR: Response rate
SLL: Small lymphocytic lymphoma
T_max_: Median time to the peak concentration
TN: Treatment naïve
VGPR: Very good partial response
WM: Waldenström macroglobulinemia
