# Supplementary material for: Chinese expert consensus on Bruton tyrosine kinase inhibitors in the treatment of B-cell malignancies
Source: Exp Hematol Oncol. 2023 Oct 16;12:92. doi: 10.1186/s40164-023-00448-5 (PMC10578030; doi:10.1186/s40164-023-00448-5)
Supplement: Supplementary file 1 — Additional file 1. Table S1. Recommendations questionnaire [file 40164_2023_448_MOESM1_ESM.docx]

**Supplementary Table 1. Recommendations questionnaire**

| **Recommendations** | **Agree** | **Disagree** |
| --- | --- | --- |
| CLL/SLL recommendation 1: BTK inhibitors are recommended in patients with CLL/SLL regardless of age, fitness, del (17p) and/or TP53 mutation, IGHV mutational status, and therapeutic settings (frontline or salvage) (Category 1). |  |  |
| CLL/SLL recommendation 2: Despite no apparent impact on decision-making for BTK inhibitor utility, tests for common prognosis biomarkers, such as del (17p) and/or TP53 mutation and IGHV mutational status, are still recommended for both scientific interests and long-term therapeutic planning after BTK inhibitor failure (Category 2A). |  |  |
| CLL/SLL recommendation 3: Acalabrutinib and zanubrutinib have a more favorable safety profile than ibrutinib, especially in terms of cardiovascular events. Based on the head-head comparison studies results, zanubrutinib could be recommended as the most preferred treatment regimen for patients with CLL/SLL (Category 1). |  |  |
| MCL recommendation 1: BTK inhibitors combined with chemoimmunotherapy are recommended in MCL patients aged ≥ 65 years or frail patients. (Category 2B) |  |  |
| MCL recommendation 2: BTK inhibitors are recommended for both suitable and unsuitable ASCT candidates during induction and as maintenance therapy (Category 2B). |  |  |
| MCL recommendation 3: BTK inhibitors are the preferred treatment choice for patients with R/R MCL (Category 2A) and are recommended to start BTK inhibitors treatment as early as possible for better outcomes (Category 2A). |  |  |
| MCL recommendation 4: Acalabrutinib and zanubrutinib have a more favorable safety profile than ibrutinib, especially in terms of cardiovascular events. Based on the head-head to comparison studies results, zanubrutinib is recommended as the most preferred treatment regimen for patients with MCL (Category 2B). |  |  |
| DLBCL recommendation 1: BTK inhibitors are recommended as an optional treatment regimen in non-GCB DLBCL patients. (Category 2B) |  |  |
| DLBCL recommendation 2: BTK inhibitors are recommended as an optional treatment regimen in DLBCL patients with specific subtypes (correlation of BCL2/MYC expression, CD79B/MYD88 mutation) (Category 2A). |  |  |
| DLBCL recommendation 3: For patients with poor response or unfit (elderly or frail) for standard chemotherapy (i.e., R-CHOP), BTK inhibitors with less chemotherapy (i.e., R-miniCHOP) or chemo-free regimen (BTK inhibitors with rituximab and/or lenalidomide) could be suggested (Category 2B). |  |  |
| CNSL recommendation 1: It is recommended that CNSL patients be treated with BTK inhibitor-based regimens, either alone or in combination with chemotherapy, as a treatment approach for induction/re-induction and maintenance therapy in both TN and R/R patients (Category 2B). |  |  |
| WM recommendation 1: BTK inhibitors as a monotherapy or in combination with rituximab are recommended for the treatment of WM (Category 1). |  |  |
| WM recommendation 2: Zanubrutinib is one of the treatment options for the treatment of patients with MYD88WT (Category 2A). |  |  |
| WM recommendation 3: Zanubrutinib is the preferred treatment option for patients with CXCR4 mutation (Category 1). |  |  |
| WM recommendation 4: Zanubrutinib is recommended as the preferred treatment regimen rather than ibrutinib considering the balance of efficacy and safety, especially for CV events in a head-to-head study (Category 1). |  |  |
| MZL recommendation 1: BTK inhibitor is recommended as one of the treatment options for patients with R/R MZL (Category 2A). |  |  |
| MZL recommendation 2: Zanubrutinib is highly recommended considering a better safety profile than first ibrutinib, especially in terms of cardiovascular events (Category 1). |  |  |
| FL recommendation 1: Zanubrutinib with obinutuzumab is recommended as one of the treatment options for the treatment of patients with R/R FL (Category 2B). |  |  |
